# Investigating the relationship between BMI across adulthood and late life brain pathologies

**DOI:** 10.1186/s13195-021-00830-7

**Published:** 2021-04-30

**Authors:** Christopher A. Lane, Josephine Barnes, Jennifer M. Nicholas, John W. Baker, Carole H. Sudre, David M. Cash, Thomas D. Parker, Ian B. Malone, Kirsty Lu, Sarah-Naomi James, Ashvini Keshavan, Sarah Buchanan, Sarah Keuss, Heidi Murray-Smith, Andrew Wong, Elizabeth Gordon, William Coath, Marc Modat, David Thomas, Rebecca Hardy, Marcus Richards, Nick C. Fox, Jonathan M. Schott

**Affiliations:** 1 Dementia Research Centre, UCL Queen Square Institute of Neurology, University College London, Box 16, Queen Square, London, WC1N 3BG UK; 2 Hoffmann-La Roche UK Ltd, London, UK; 3 Department of Medical Statistics, London School of Hygiene and Tropical Medicine, University of London, London, UK; 4 School of Biomedical Engineering and Imaging Sciences, King’s College London, London, UK; 5 MRC Unit for Lifelong Health and Ageing at UCL, London, UK; 6 Leonard Wolfson Experimental Neurology Centre, UCL Queen Square Institute of Neurology, University College London, London, UK; 7 Neuroradiological Academic Unit, Department of Brain Repair and Rehabilitation, UCL Queen Square Institute of Neurology, University College London, London, UK; 8 UK Dementia Research Institute at UCL, University College London, London, UK

**Keywords:** Cohort studies, Epidemiology, MRI, PET, Alzheimer’s disease

## Abstract

**Background:**

In view of reported associations between high adiposity, particularly in midlife and late-life dementia risk, we aimed to determine associations between body mass index (BMI), and BMI changes across adulthood and brain structure and pathology at age 69–71 years.

**Methods:**

Four hundred sixty-five dementia-free participants from Insight 46, a sub-study of the British 1946 birth cohort, who had cross-sectional T1/FLAIR volumetric MRI, and florbetapir amyloid-PET imaging at age 69–71 years, were included in analyses. We quantified white matter hyperintensity volume (WMHV) using T1 and FLAIR 3D-MRI; β-amyloid (Aβ) positivity/negativity using a SUVR approach; and whole brain (WBV) and hippocampal volumes (HV) using 3D T1-MRI. We investigated the influence of BMI, and BMI changes at and between 36, 43, 53, 60–64, 69 and 71 years, on late-life WMHV, Aβ-status, WBV and mean HV. Analyses were repeated using overweight and obese status.

**Results:**

At no time-point was BMI, change in BMI or overweight/obese status associated with WMHV or WBV at age 69–71 years. Decreasing BMI in the 1–2 years before imaging was associated with an increased odds of being β-amyloid positive (OR 1.45, 95% confidence interval 1.09, 1.92). There were associations between being overweight and larger mean HV at ages 60–64 (*β* = 0.073 ml, 95% CI 0.009, 0.137), 69 (*β* = 0.076 ml, 95% CI 0.012, 0.140) and 71 years (*β* = 0.101 ml, 95% CI 0.037, 0.165). A similar, albeit weaker, trend was seen with obese status.

**Conclusions:**

Using WMHV, β-amyloid status and brain volumes as indicators of brain health, we do not find evidence to explain reported associations between midlife obesity and late-life dementia risk. Declining BMI in later life may reflect preclinical Alzheimer’s disease.

**Supplementary Information:**

The online version contains supplementary material available at 10.1186/s13195-021-00830-7.

## Background

Dementia affects 44 million people worldwide, with prevalence predicted to triple by 2050. Vascular risk factors, including obesity, have been implicated as potential targets for intervention to reduce dementia risk. However, it remains unclear how obesity might influence subsequent brain health, and whether there are sensitive age periods when risk exposure is particularly damaging.

Positive associations have been reported between obesity and all-cause dementia [1, 2], but also with clinically-diagnosed vascular [3, 4] and Alzheimer’s disease (AD) dementia [5–7] with more consistent findings relating to midlife, rather than late life, adiposity [8–10]. The pathophysiological mechanisms mediating these relationships are not well understood. Associations may be driven by downstream consequences of obesity on other vascular risk factors, namely hypertension, insulin resistance, and dyslipidaemia [11], which have all been independently implicated in dementia risk. Obesity also influences cardiovascular health via endothelial dysfunction and pro-inflammatory routes [12], which might also be implicated in cerebral health.

We sought to determine the relationship between body mass index (BMI), longitudinal BMI changes, and obesity status across adulthood and cerebral small vessel disease (SVD), brain volumes and fibrillar β-amyloid pathology in early late life. We studied individuals from a British birth cohort who have had BMI prospectively and serially measured from their mid-30s onwards, and cerebral imaging aged 69–71 years. We hypothesised that (1) BMI would be most strongly associated with an imaging marker of cerebral SVD and (2) there would be specific periods when BMI and changes in BMI would influence brain pathology.

## Methods

### Study design and participants

Participants were from Insight 46, a sub-study of the MRC National Survey of Health and Development (NSHD), a birth cohort which initially comprised 5362 individuals born throughout mainland Britain in 1 week in 1946 [13]. Follow-up has included > 20 contacts since birth, including home assessments by research nurses at ages 36, 43, 53, and 69 years, and assessment at a clinical research facility at age 60–64 years. Participants were eligible for inclusion in the Insight 46 sub-study if this defined set of life course data were available, and where willingness to attend a clinic visit in London had previously been expressed. Individuals with contraindications to MRI or PET (including claustrophobia and metallic implants) were excluded. Further eligibility criteria have been described elsewhere [14]. Between 2015 and 2018, 502 participants were assessed at University College London [14], when aged 69–71 years. An overview of recruitment is provided in Figure e-1 (supplementary). Comparisons between Insight 46 participants and the larger NSHD have previously been reported [15].

### Procedures

Imaging was performed on a single Biograph mMR 3 T PET/MRI scanner (Siemens Healthcare, Erlangen), with simultaneous acquisition of dynamic PET/MR data, including volumetric (1.1 mm isotropic) T1 and T2-weighted Fluid Attenuated Inversion Recovery (FLAIR) sequences [14]. β-amyloid burden was assessed using [16] F florbetapir (Amyvid). PET data were processed using an automated in-house processing pipeline including pseudo-CT attenuation correction [14]. Global standardised uptake value ratio (SUVR) was calculated from cortical regions of interest (ROIs) comprising the lateral and medial frontal, anterior and posterior cingulate, lateral parietal, and lateral temporal regions, normalised to eroded subcortical white matter. Positive/negative β-amyloid status was determined using a Gaussian mixture model applied to SUVR values, taking the 99th percentile of the lower (β-amyloid negative) Gaussian as the cut-point (0.61).

Volumetric T1-weighted and FLAIR images underwent visual quality control (QC), before processing using validated automated pipelines: [14] whole-brain volume (WBV) segmentation using MAPS [17], hippocampal volumes (HV) using STEPS [18], with appropriate manual editing; and total intracranial volume (TIV) using SPM12 [16]. A validated, unsupervised automated algorithm, BaMoS (Bayesian Model Selection) [19] was used to segment white matter hyperintensities (WMH) from T1/FLAIR images, followed by visual QC and manual editing where appropriate, generating a global WMH volume (WMHV) including subcortical grey matter but excluding infratentorial regions.

Participants were classified as cognitively normal, having mild cognitive impairment (according to research criteria [20]) or dementia, based on expert consensus, informed by clinical history, informant history, MMSE [21], and cognitive performance (WMS-R Logical Memory test [22] and WAIS-R Digit symbol substitution test [23]).

### BMI and other covariates

Height and weight measurements were collected by standard protocol as part of NSHD assessments at ages 36, 43, 53, 60–64, and 69 years, and at the Insight 46 assessment at age 69–71 years (which for clarity will subsequently be referred to as 71 years). BMI was defined as the weight in kilogrammes divided by the square of the height (in metres). Abdominal circumference (AC), was measured to the nearest mm using a standardised protocol at 36, 43, 53, 60–64 and 69 years.

Using an approach previously employed in the NSHD, BMI change for the periods 36–43, 43–53, 53–60/64, 60/64–69 and 69–71 years, conditional on earlier measurements, was calculated as the residual from the regression of each BMI measure (from 43 years) on the earlier measure(s) for each sex, using individuals with available data at all time-points. Residuals represent a change in BMI above/below that expected on average given the earlier BMI. Residuals were standardised, allowing comparison between periods [24]. AC change variables were derived using the same approach.

Vascular risk factors selected for adjustment in statistical models included systolic blood pressure (SBP) contemporaneous with BMI measurement, and smoking status, hypercholesterolaemia, and diabetes mellitus (DM) status at the time of the Insight 46 assessment. Seated BP was measured in the upper arm twice after 5 min’ rest at ages 36, 43, 53, 60–64 and 69, and a lying BP collected after 3 min rest at age 71 years. The second BP measure was used for analyses, unless missing. Smoking status was defined by questionnaire (at age 68 years, or if missing at 60–64 years) as never-smoked, ex-smoker and current smoker. Hypercholesterolaemia status was based on self-reported use of cholesterol-lowering medication at Insight 46 assessment or random total cholesterol ≥ 5 mmol/L at 69 years. Diabetes mellitus (DM) status was based on self-reported diabetic medication use at Insight 46 assessment, HbA1c > 6.5% or a self-reported diagnosis at 69 years. APOE genotyping was performed using standard techniques and individuals categorised as APOE-ε4 carriers or non-carriers. Adult socioeconomic position (SEP) was defined as non-manual or manual, based on the occupation at 53 years according to the UK Registrar General’s Classification of Occupations.

A measure of affective symptoms was available at age 69 years derived from the general health questionnaire (GHQ-28 [25]), with a score of > 5 defined as probable anxiety or depression.

### Statistical analysis

Analyses were performed in Stata v.14.1 (Stata Corp). Participants were included if they were dementia-free, and had acceptable quality amyloid PET/MR imaging. For WMH and brain volume analyses, individuals with cortical infarcts inappropriately segmented as WMH (*n* = 5), atypical vascular pathology (*n* = 1) or white matter pathologies not considered to be of vascular origin e.g. demyelination (*n* = 3) were excluded. For brain volume analysis individuals also needed a useable amyloid scan, since the amyloid status was included as a covariate in the fully-adjusted model. Otherwise, all individuals, including those with neurological diagnoses, with available BMI/AC data at any time-point, were included for generalisability.

BMI and AC at each visit (up to age 69 years, which was the last time-point that an assessment was performed across the entire cohort) were compared between Insight 46 participants and the larger NSHD, using unadjusted linear mixed effect models for men and women separately, using all available measurements. An unstructured residual variance-covariance matrix was used to model the correlation between repeated measures in an individual.

Due to the non-normal distribution of WMHV, generalised linear models (GLM) using the gamma distribution with log link were used to investigate relationships between BMI at each age separately and WMHV at 71 years. Linear regression was used to investigate relationships between BMI at each age and WBV and mean HV at 71 years. Model 1 adjusted for sex, TIV and scanning age. Model 2 also adjusted for SBP contemporaneous with BMI measurement. Model 3 additionally adjusted for other potential cardiovascular confounders: smoking status, diabetic status, hypercholesterolaemia status at the time of Insight 46 assessment, and adult SEP. For brain volume analyses, to explore BMI influences independently of measurable brain pathologies, model 3 also adjusted for global WMHV and β-amyloid status.

BMI change variables were then treated as the main predictor within GLM/linear regression models: Model 1(c): all BMI conditional change variables included and adjusted for sex, TIV and scanning age. Model 2(c): each conditional change variable assessed individually and adjusted for contemporaneous SBP (e.g. in the model examining BMI change between 36 and 43 years, SBP at 43 years was included in the model) and the covariates described in model 3 above. These models address whether, regardless of previous weight gain, there is a period when weight change has a particularly strong association with an imaging outcome measure at age 71 years.

We used logistic regression to test associations between BMI at ages 36 through to 71 years and β-amyloid status (positive or negative). Model 1 adjusted for sex. Model 2 further adjusted for *APOE*-ε4 status. Model 3 also adjusted for contemporaneous SBP. Additional vascular risk factors were not included in models due to the limited number of β-amyloid-positive individuals. Associations between BMI change and β-amyloid status were investigated using two models: Model 1(c) included all conditional change variables within the same model, adjusting for sex. Model 2(c) assessed each conditional change variable individually and adjusted for contemporaneous SBP and *APOE*-ε4 status. A differential influence of BMI (or change) on β-amyloid status by *APOE*-ε4 status was tested using an interaction term in fully-adjusted models.

In an exploratory analysis, all continuous BMI and BMI change models were repeated replacing BMI with AC, to determine whether a measure of central adiposity might be more strongly associated with imaging measures.

Model assumptions were checked with regression diagnostics, including checks of linearity by examination of residuals. Possible non-linear relationships were explored through the creation of a categorical variable defining individuals as normal weight (BMI < 25 kg/m^2^), overweight (25 < BMI < 30 kg/m^2^) or obese (BMI > 30 kg/m^2^) which was then used in models 1–3, replacing BMI as the independent variable. Too few individuals were underweight (BMI < 18.5 kg/m^2^) at any given age to create a separate category and were therefore treated as normal weight. Sensitivity analyses were performed excluding these individuals. Interactions between BMI and sex at each time point were investigated with appropriate interaction terms.

To explore the potential influence of cumulative adiposity on imaging measures, individuals were categorised by obese status at each time point and then a cumulative score calculated using the sum of the number of time-points that an individual was classed as obese. This variable was used as the predictor of interest in fully-adjusted models for each imaging outcome. Individuals missing any BMI measures were excluded from this analysis.

### Standard protocol approvals, registrations, and patient consents

Ethical approvals for the wider NSHD have been described [26]. Insight 46 was approved by the Queen Square Research Ethics Committee. All participants provided written informed consent.

### Data availability policy

A data-sharing policy is in place: anonymised data will be shared by request from any qualified investigator (https://skylark.ucl.ac.uk/NSHD/doku.php).

## Results

Of the 502 individuals assessed as part of Insight 46, 471 (93.4%) completed the imaging protocol. 468 (93.2%) of Insight 46 individuals were dementia-free. Following imaging processing and QC, 457 (91.0%) scans were available for amyloid analysis, 445 (88.6%) for brain volume analysis and 453 (90.2%) for WMHV analysis. Participant characteristics of those with available imaging are summarised in Table 1. Table e-1 (supplementary) summarises participant characteristics between individuals with complete BMI data, individuals missing BMI data at any time-point and individuals who did not complete the scanning protocol. Compared with individuals who completed scanning, participants who did not complete scanning tended to have a higher BMI (age 71 years: mean BMI (SD) 27.4 (4.2) kg/m^2^ in scanned individuals versus 30.4 (5.7) kg/m^2^ in individuals not scanned).

**Table 1:** Characteristics of dementia-free participants having at least one outcome of interest

Variable		***N*** men/women (total)	Value in men and women combined
**Age at assessment, mean (SD)**		238/227 (465)	70.7 (0.7)
**Age at scanning, mean (SD)**		238/227 (465)	70.7 (0.7)
**β-Amyloid positive,** ***n*** **(%)**		232/225 (457)	83 (18.2)
**Whole brain volume in ml, mean (SD)**		227/218 (445)	1100.0 (98.4)
**Mean hippocampal volume in ml, mean (SD)**		227/218 (445)	3.1 (0.3)
**White matter hyperintensity volume in ml, median (IQR)**		233/220 (453)	3.1 (1.6, 6.8)
**Total intracranial volume in ml, mean (SD)**		233/220 (453)	1434.0 (132.3)
**MMSE/30, mean (SD)**		238/227 (465)	29.3 (0.9)
**BMI at 36 years in kg/m**^**2**^**, mean (SD)**		217/209 (426)	23.7 (3.1)
**BMI at 43 years in kg/m**^**2**^**, mean (SD)**		231/215 (446)	24.9 (3.2)
**BMI at 53 years in kg/m**^**2**^**, mean (SD)**		232/224 (456)	26.9 (4.1)
**BMI at 60–64 years in kg/m**^**2**^**, mean (SD)**		238/227 (465)	27.5 (4.1)
**BMI at 69 years in kg/m**^**2**^**, mean (SD)**		236/222 (458)	27.6 (4.4)
**BMI at 71 years in kg/m**^**2**^**, mean (SD)**		238/227 (465)	27.6 (4.4)
**AC at 36 years in cm, mean (SD)**		217/209 (426)	81.7 (11.0)
**AC at 43 years in cm, mean (SD)**		230/218 (448)	83.0 (11.2)
**AC at 53 years in cm, mean (SD)**		232/225 (457)	90.4 (12.0)
**AC at 60–64 years in cm, mean (SD)**		238/227 (465)	95.5 (11.7)
**AC at 69 years in cm, mean (SD)**		236/223 (459)	95.1 (12.3)
**Weight category at age 36, n (%)**	**Underweight**	217/209 (426)	9 (2.1)
	**Normal weight**		303 (71.1)
	**Overweight**		101 (23.7)
	**Obese**		13 (3.1)
**Weight category at age 43,** ***n*** **(%)**	**Underweight**	231/215 (446)	3 (0.7)
	**Normal weight**		245 (54.9)
	**Overweight**		168 (37.7)
	**Obese**		30 (6.7)
**Weight category at age 53,** ***n*** **(%)**	**Underweight**	232/224 (456)	0 (0)
	**Normal weight**		155 (34.0)
	**Overweight**		220 (48.3)
	**Obese**		81 (17.8)
**Weight category at age 60–64,** ***n*** **(%)**	**Underweight**	238/227 (465)	0 (0)
	**Normal weight**		143 (30.8)
	**Overweight**		201 (43.2)
	**Obese**		121 (26.0)
**Weight category at age 69,** ***n*** **(%)**	**Underweight**	236/222 (458)	2 (0.4)
	**Normal weight**		142 (31.0)
	**Overweight**		195 (42.6)
	**Obese**		119 (26.0)
**Weight category at age 71,** ***n*** **(%)**	**Underweight**	238/227 (465)	2 (0.4)
	**Normal weight**		145 (31.2)
	**Overweight**		195 (41.9)
	**Obese**		123 (26.5)
**Smoking status at age 68,** ***n*** **(%)**	**Current smoker**	238/227 (465)	16 (3.4)
	**Ex-smoker**		286 (61.5)
	**Never smoked**		163 (35.1)
**Hypercholesterolaemia at age 71,** ***n*** **(%)**		238/227 (465)	369 (79.4)
**Diabetes mellitus at age 71,** ***n*** **(%)**		237/224 (461)	50 (10.9)
**SBP at age 71 in mmHg, mean (SD)**		237/227 (464)	137.0 (16.9)
**Adult socioeconomic position,** ***n*** **(%)**	**Non-manual (Class I-IIIN)**	238/2427 (465)	395 (84.9)
	**Manual (Class IIIM-V)**		70 (15.1)
***APOE*-ε4 carrier (1 or 2 alleles),** ***n*** **(%)**		230/225 (455)	131 (28.8)

*BMI* body mass index, *IQR* interquartile range, *MMSE* mini-mental state examination, *n* number, *SBP* systolic blood pressure, *SD* standard deviation

**Fig. 1:**
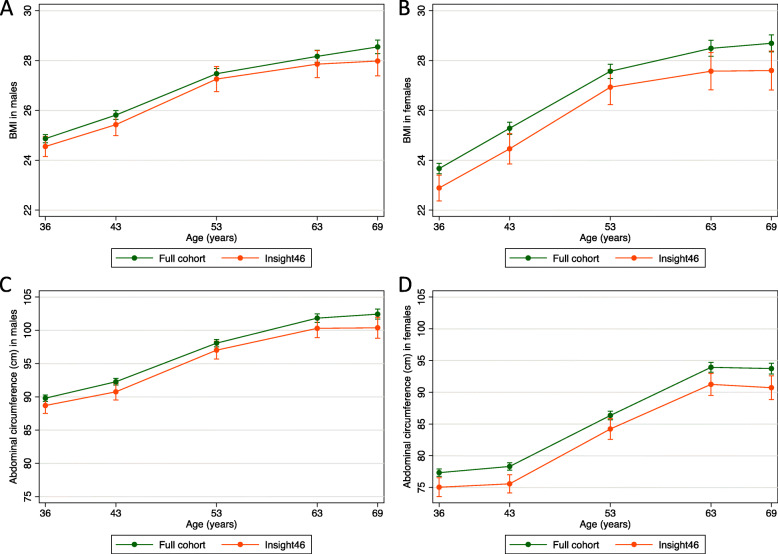
BMI and AC across adulthood in Insight 46 participants and the larger NSHD Line graphs comparing predicted BMI (top panels) and AC (bottom panels) in males (**a**, **c**) and females (**b**, **d**) between Insight 46 participants and individuals with available measures in the larger NSHD from ages 36 up to 69 years (the last age the whole NSHD cohort were assessed). 95% confidence intervals shown. Predictions are marginal means at each time-point from a linear mixed effects model fitted jointly across BMI or AC measures at all time-points. AC, abdominal circumference; BMI, body mass index

Mean age at scanning was 70.7; SD 0.7 years. 18.2% were β-amyloid positive. Prevalence of obesity increased from 3.1% at age 36 years to 26.5% at age 71 years. Figure 1 shows the pattern of BMI and AC trajectories in Insight 46 individuals (*n* = 502) compared with the larger NSHD (BMI: *n* = 3188; AC: *n* = 3193). Female participants in Insight 46 had a predicted BMI 0.8 kg/m^2^ (*p* = 0.03) and AC 2.5 cm smaller (*p* = 0.01) than those in the main cohort at any given time-point. Differences in males were smaller, with those in Insight 46 having a predicted BMI 0.3 kg/m^2^ (*p* = 0.2) less and AC 1.2 cm (*p* = 0.2) smaller than those in the main cohort.

BMI, BMI change and weight status at all time-points investigated were not associated with WBV or WMHV at age 71 years (Tables 2, 3 and 4).

**Table 2:** Associations between BMI and weight across adulthood, and WMHV and β-amyloid status at age 71

		Relative change in WMHV (95% CI)			Adjusted OR for β-amyloid positivity (95% CI)		
		Model 1	Model 2	Model 3	Model 1	Model 2	Model 3
**Age 36** WMHV: Model 1: *n* = 415 Model 2: *n* = 411 Model 3: *n* = 409 Amyloid: Model 1: *n* = 418 Model 2: *n* = 416 Model 3: *n* = 412	**BMI**	0.99 (0.95, 1.02)	0.99 (0.95, 1.02)	0.98 (0.94, 1.02)	0.99 (0.91, 1.08)	0.98 (0.89, 1.08)	0.98 (0.89, 1.08)
	**Normal weight**	REF	REF	REF	REF	REF	REF
	**Overweight**	1.01 (0.78, 1.32)	1.01 (0.78, 1.30)	0.95 (0.73, 1.23)	1.39 (0.78, 2.49)	1.26 (0.68, 2.33)	1.23 (0.67, 2.29)
	**Obese**	0.99 (0.54, 1.82)	0.97 (0.53, 1.78)	0.95 (0.52, 1.74)	0.41 (0.05, 3.21)	0.50 (0.06, 4.14)	0.48 (0.06, 4.02)
**Age 43** WMHV: Model 1: *n* = 434 Model 2: *n* = 428 Model 3: *n* = 424 Amyloid: Model 1: *n* = 438 Model 2: *n* = 436 Model 3: *n* = 430	**BMI**	1.01 (0.97, 1.04)	1.00 (0.97, 1.04)	1.00 (0.96, 1.03)	1.00 (0.93, 1.08)	0.99 (0.92, 1.08)	0.99 (0.91, 1.08)
	**Normal weight**	REF	REF	REF	REF	REF	REF
	**Overweight**	1.05 (0.84, 1.31)	1.03 (0.83, 1.29)	1.01 (0.81, 1.26)	0.70 (0.41, 1.21)	0.73 (0.41, 1.28)	0.72 (0.41, 1.28)
	**Obese**	1.03 (0.68, 1.56)	0.95 (0.62, 1.45)	0.94 (0.61, 1.45)	1.26 (0.51, 3.13)	1.21 (0.47, 3.12)	1.26 (0.49, 3.29)
**Age 53** WMHV: Model 1: *n* = 444 Model 2: *n* = 440 Model 3: *n* = 436 Amyloid: Model 1: *n* = 448 Model 2: *n* = 446 Model 3: *n* = 441	**BMI**	1.00 (0.98, 1.03)	1.00 (0.97, 1.02)	0.99 (0.97, 1.02)	0.98 (0.92, 1.04)	0.97 (0.91, 1.03)	0.98 (0.92, 1.05)
	**Normal weight**	REF	REF	REF	REF	REF	REF
	**Overweight**	1.18 (0.94, 1.48)	1.13 (0.90, 1.41)	1.10 (0.87, 1.38)	**0.56 (0.33, 0.97)**	**0.49 (0.27, 0.88)**	**0.51 (0.29, 0.92)**
	**Obese**	0.98 (0.73, 1.31)	0.93 (0.69, 1.25)	0.90 (0.66, 1.22)	0.85 (0.44, 1.67)	0.73 (0.36, 1.48)	0.89 (0.43, 1.87)
**Age 60–64** WMHV: Model 1: *n* = 453 Model 2: *n* = 452 Model 3: *n* = 448 Amyloid: Model 1: *n* = 457 Model 2: *n* = 455 Model 3: *n* = 454	**BMI**	1.01 (0.98, 1.03)	1.01 (0.98, 1.03)	1.00 (0.98, 1.03)	0.96 (0.90, 1.02)	0.95 (0.89, 1.01)	0.95 (0.89, 1.02)
	**Normal weight**	REF	REF	REF	REF	REF	REF
	**Overweight**	1.11 (0.87, 1.42)	1.11 (0.88, 1.42)	1.09 (0.86, 1.40)	0.89 (0.51, 1.55)	0.87 (0.48, 1.56)	0.88 (0.49, 1.59)
	**Obese**	1.17 (0.89, 1.54)	1.15 (0.88, 1.50)	1.10 (0.83, 1.45)	0.68 (0.36, 1.31)	0.66 (0.33, 1.31)	0.70 (0.35, 1.40)
**Age 69** WMHV: Models 1, 2 and 3: *n* = 446 Amyloid: Model 1: *n* = 450 Models 2 and 3: *n* = 448	**BMI**	1.00 (0.98, 1.03)	1.00 (0.98, 1.03)	1.00 (0.97, 1.02)	0.96 (0.90, 1.01)	0.95 (0.90, 1.01)	0.95 (0.89, 1.01)
	**Normal weight**	REF	REF	REF	REF	REF	REF
	**Overweight**	1.01 (0.79, 1.29)	1.02 (0.80, 1.29)	1.01 (0.79, 1.28)	0.76 (0.44, 1.32)	0.78 (0.44, 1.40)	0.78 (0.44, 1.40)
	**Obese**	1.00 (0.77, 1.32)	0.99 (0.76, 1.29)	0.97 (0.74, 1.26)	0.57 (0.29, 1.09)	0.55 (0.28, 1.10)	0.54 (0.27, 1.09)
**Age 71** WMHV: Model 1: *n* = 453 Model 2: *n* = 452 Model 3: *n* = 448 Amyloid: Model 1: *n* = 457 Model 2: *n* = 455 Model 3: *n* = 454	**BMI**	1.01 (0.98, 1.03)	1.00 (0.98, 1.03)	1.00 (0.97, 1.02)	0.95 (0.90, 1.01)	0.94 (0.89, 1.00)	0.95 (0.89, 1.01)
	**Normal weight**	REF	REF	REF	REF	REF	REF
	**Overweight**	1.15 (0.90, 1.47)	1.14 (0.89, 1.45)	1.12 (0.88, 1.43)	0.92 (0.53, 1.60)	0.86 (0.48, 1.53)	0.89 (0.49, 1.60)
	**Obese**	1.16 (0.89, 1.53)	1.12 (0.86, 1.47)	1.07 (0.82, 1.41)	0.58 (0.30, 1.13)	0.55 (0.27, 1.11)	0.57 (0.28, 1.17)

WMHV coefficients represent the relative change in mean WMHV per 1 unit change in BMI or change in weight status using normal weight as the reference group, using GLM. Adjusted β-amyloid ORs are quoted per 1 unit change in BMI or change in weight status using normal weight as the reference group, using logistic regression models. 95% confidence intervals are also shown. Associations significant at *p* < 0.05 are highlighted in bold. Model numbers are stated in the left-hand column. WMHV models: Model 1 adjusted for TIV, sex and age at scanning; Model 2 also adjusted for contemporaneous SBP; Model 3 also adjusted for adult SEP, DM, hypercholesterolaemia, and smoking status. β-amyloid models: Model 1 adjusted for sex. Model 2 also adjusted for APOE-ε4 status. Model 3 also adjusted for contemporaneous SBP. *BMI* body mass index, *CI* confidence interval, *DM* diabetes mellitus, *GLM* generalised linear model, *REF* reference, *SBP* systolic blood pressure, *SEP* socioeconomic position, *TIV* total intracranial volume, *WMHV* white matter hyperintensity volume

**Table 3:** Associations between BMI and weight across adulthood, and WBV and mean HV at age 71

		WBV ***β*** coefficient (95% CI)			Mean HV ***β*** coefficient (95% CI)		
		Model 1	Model 2	Model 3	Model 1	Model 2	Model 3
**Age 36** Model 1: *n* = 407 Model 2: *n* = 403 Model 3: *n* = 401	**BMI**	1.1 (− 0.4, 2.5)	1.0 (− 0.4, 2.5)	1.3 (− 0.2, 2.7)	**0.011 (0.002, 0.020)**	**0.010 (0.001, 0.020)**	0.009 (0, 0.019)
	**Normal weight**	REF	REF	REF	REF	REF	REF
	**Overweight**	1.15 (− 9.5, 11.8)	1.2 (− 9.5, 11.9)	2.2 (−8.4, 12.9)	0.031 (− 0.036, 0.099)	0.028 (− 0.039, 0.095)	0.029 (− 0.040, 0.097)
	**Obese**	18.8 (−6.1, 43.6)	19.1 (−5.9, 44.0)	21.9 (−3.0, 46.8)	**0.192 (0.034, 0.349)**	**0.188 (0.031, 0.345)**	**0.167 (0.007, 0.327)**
**Age 43** Model 1: *n* = 426 Model 2: *n* = 420 Model 3: *n* = 416	**BMI**	1.0 (− 0.4, 2.3)	1.1 (− 0.3, 2.4)	1.1 (− 0.2, 2.5)	0.006 (− 0.002, 0.015)	0.006 (− 0.003, 0.015)	0.004 (− 0.005, 0.013)
	**Normal weight**	REF	REF	REF	REF	REF	REF
	**Overweight**	6.0 (− 3.2, 15.2)	6.5 (− 2.8, 15.7)	7.6 (−1.6, 16.9)	0.041 (− 0.017, 0.100)	0.044 (− 0.015, 0.103)	0.035 (− 0.025, 0.094)
	**Obese**	16.5 (− 0.5, 33.6)	17.1 (− 0.2, 34.4)	16.8 (− 0.6, 34.2)	0.072 (− 0.037, 0.180)	0.057 (− 0.053, 0.167)	0.047 (− 0.065, 0.159)
**Age 53** Model 1: *n* = 436 Model 2: *n* = 432 Model 3: *n* = 428	**BMI**	0.3 (− 0.7, 1.3)	0.6 (− 0.4, 1.7)	0.8 (− 0.2, 1.9)	0.005 (− 0.002, 0.011)	0.006 (− 0.001, 0.013)	0.005 (− 0.003, 0.012)
	**Normal weight**	REF	REF	REF	REF	REF	REF
	**Overweight**	− 2.3 (− 11.8, 7.3)	− 0.6 (− 10.2, 9.0)	0.3 (− 9.4, 9.9)	0.008 (− 0.054, 0.069)	0.013 (− 0.049, 0.075)	− 0.001 (− 0.065, 0.062)
	**Obese**	6.3 (−6.1, 18.7)	9.6 (−3.2, 22.5)	11.1 (− 1.8, 24.0)	0.062 (− 0.017, 0.142)	0.079 (− 0.004, 0.162)	0.063 (− 0.021, 0.148)
**Age 60–64** Model 1: *n* = 445 Model 2: *n* = 444 Model 3: *n* = 440	**BMI**	− 0.2 (− 1.2, 0.8)	− 0.1 (− 1.1, 0.9)	0.2 (− 0.9, 1.2)	0.005 (− 0.001, 0.012)	0.006 (0, 0.013)	0.005 (− 0.002, 0.011)
	**Normal weight**	REF	REF	REF	REF	REF	REF
	**Overweight**	− 0.5 (− 10.4, 9.4)	0.2 (− 9.7, 10.2)	0.5 (− 9.5, 10.4)	**0.077 (0.014, 0.140)**	**0.083 (0.019, 0.147)**	**0.073 (0.009, 0.137)**
	**Obese**	−2.6 (−13.7, 8.4)	−1.3 (− 12.5, 10.0)	− 0.2 (− 11.7, 11.4)	0.066 (− 0.005, 0.136)	**0.076 (0.005, 0.148)**	0.059 (− 0.015, 0.134)
**Age 69** Models 1, 2 and 3: *n* = 438	**BMI**	− 0.3 (− 1.2, 0.7)	− 0.2 (− 1.2, 0.7)	0.0 (−1.0, 1.0)	0.005 (− 0.002, 0.011)	0.005 (− 0.001, 0.011)	0.004 (− 0.002, 0.010)
	**Normal weight**	REF	REF	REF	REF	REF	REF
	**Overweight**	1.6 (− 8.3, 11.6)	1.7 (− 8.3, 11.6)	3.4 (− 6.5, 13.4)	**0.077 (0.013, 0.140)**	**0.077 (0.013, 0.140)**	**0.076 (0.012, 0.140)**
	**Obese**	− 3.4 (−14.5, 7.7)	−2.5 (− 13.8, 8.7)	0.1 (− 11.2, 11.3)	0.046 (− 0.025, 0.116)	0.049 (− 0.022, 0.121)	0.040 (− 0.033, 0.112)
**Age 71** Model 1: *n* = 445 Model 2: *n* = 444 Model 3: *n* = 440	**BMI**	− 0.2 (− 1.2, 0.7)	− 0.1 (− 1.1, 0.8)	0.0 (− 0.9, 1.0)	0.005 (− 0.001, 0.011)	0.006 (0, 0.012)	0.004 (− 0.002, 0.010)
	**Normal weight**	REF	REF	REF	REF	REF	REF
	**Overweight**	3.6 (−6.2, 13.5)	4.9 (−5.0, 14.8)	4.5 (−5.4, 14.4)	**0.097 (0.034, 0.159)**	**0.108 (0.045, 0.171)**	**0.101 (0.037, 0.165)**
	**Obese**	− 4.3 (−15.3, 6.7)	− 3.0 (− 14.1, 8.0)	− 1.6 (− 12.8, 9.7)	**0.078 (0.009, 0.148)**	**0.090 (0.020, 0.161)**	**0.076 (0.003, 0.149)**

*β*-coefficients represent the change in brain volume (ml) per 1 unit change in BMI or change in weight status using normal weight as the reference group. 95% confidence intervals are also shown. Associations significant at *p* < 0.05 are highlighted in bold. Model numbers are stated in the left-hand column. Model 1 adjusted for TIV, sex and age at scanning; Model 2 also adjusted for contemporaneous SBP; Model 3 also adjusted for adult SEP, DM, hypercholesterolaemia, smoking status, β-amyloid status and global WMHV. *BMI* body mass index, *CI* confidence interval, *DM* diabetes mellitus, *HV* hippocampal volume, *REF* reference, *SBP* systolic blood pressure, *SEP* socioeconomic position, *TIV* total intracranial volume, *WBV* whole brain volume, *WMHV* white matter hyperintensity volume

**Table 4:** Associations between BMI change and global WMHV, β-amyloid status and brain volumes at age 71

	Relative change in WMHV (95% CI)		β-amyloid adjusted OR (95% CI)		WBV ***β*** coefficient (95% CI)		Mean HV ***β*** coefficient (95% CI)	
	Model 1(c)	Model 2(c)	Model 1(c)	Model 2(c)	Model 1(c)	Model 2(c)	Model 1(c)	Model 2(c)
**36–43 years** WMHV: Model 1: *n* = 391 Model 2: *n* = 386 Amyloid: Model 1: *n* = 394 Model 2: *n* = 387 WBV/HV: Model 1: *n* = 383 Model 2: *n* = 378	1.08 (0.96, 1.21)	1.06 (0.94, 1.19)	1.08 (0.83, 1.41)	1.09 (0.83, 1.42)	0.9 (− 3.6, 5.5)	1.9 (− 2.8, 6.5)	− 0.006 (− 0.035, 0.023)	− 0.010 (− 0.039, 0.020)
**43–53 years** WMHV: Model 1: *n* = 391 Model 2: *n* = 387 Amyloid: Model 1: *n* = 394 Model 2: *n* = 387 WBV/HV: Model 1: *n* = 383 Model 2: *n* = 379	1.02 (0.91, 1.14)	0.99 (0.89, 1.11)	0.95 (0.74, 1.21)	0.95 (0.73, 1.25)	− 3.9 (− 8.3, 0.5)	− 1.5 (− 6.1, 3.0)	− 0.006 (− 0.033, 0.022)	0 (− 0.029, 0.029)
**53–60/64 years** WMHV: Model 1: *n* = 391 Model 2: *n* = 390 Amyloid: Model 1: *n* = 394 Model 2: *n* = 391 WBV/HV: Model 1: *n* = 383 Model 2: *n* = 382	1.06 (0.95, 1.18)	1.04 (0.93, 1.15)	0.85 (0.65, 1.11)	0.87 (0.66, 1.16)	− 4.2 (− 8.8, 0.4)	− 4.2 (− 8.7, 0.4)	0.009 (− 0.020, 0.038)	0.010 (− 0.019, 0.039)
**60/64–69 years** WMHV: Models 1 and 2: *n* = 391 Amyloid: Model 1: *n* = 394 Model 2: *n* = 392 WBV/HV: Models 1 and 2: *n* = 383	1.01 (0.91, 1.13)	0.99 (0.89, 1.11)	0.92 (0.71, 1.19)	0.93 (0.72, 1.20)	− 2.4 (− 6.8, 2.0)	− 2.4 (− 6.9, 2.1)	− 0.004 (− 0.032, 0.024)	− 0.006 (− 0.034, 0.023)
**69–71 years** WMHV: Model 1: *n* = 391 Model 2: *n* = 390 Amyloid: Model 1: *n* = 394 Model 2: *n* = 391 WBV/HV: Model 1: *n* = 383 Model 2: *n* = 382	1.01 (0.91, 1.11)	1.00 (0.90, 1.11)	**0.75 (0.57, 0.97)**	**0.69 (0.52, 0.92)**	0.1 (− 4.5, 4.6)	− 0.2 (− 4.7, 4.3)	0.002 (− 0.026, 0.031)	0.002 (− 0.027, 0.030)

WMHV coefficients represent the relative change in mean WMHV per 1 SD increase in the expected BMI change across each time-interval using GLM. Adjusted amyloid ORs are quoted per 1 SD increase in the expected BMI change from logistic regression models. Brain volume β coefficients represent the change in brain volume (ml) per 1 SD increase in the expected BMI change from linear regression models. Associations significant at *p* < 0.05 are highlighted in bold. All model 1(c) analyses include all change variables in a single model and adjust for sex (β-amyloid analyses), TIV and scanning age (WMHV and brain volume analyses). Model 2(c) analyses examine each change variable separately and also adjust for contemporaneous SBP, DM, hypercholesterolaemia, adult SEP, smoking (WMHV models) and also β-amyloid status and global WMHV (brain volume analyses). β-amyloid model 2(c) analyses account for sex, contemporaneous SBP and APOE-ε4 status. *BMI* body mass index, *CI* confidence interval, *DM* diabetes mellitus, *GLM* generalised linear model, *HV* hippocampal volume, *OR* odds ratio, *SBP* systolic blood pressure, *SD* standard deviation, *SEP* socioeconomic position, *TIV* total intracranial volume, *WBV* whole brain volume, *WMHV* white matter hyperintensity volume

At age 53 years, being overweight, compared with normal weight, was associated with a significantly lower risk of being β-amyloid positive at age 71 years (OR 0.51, *p* = 0.026, model 3). An association in the same direction, although substantially weaker, was seen with obese status (OR 0.89, *p* = 0.77, model 3). This pattern persisted at subsequent time-points, albeit associations were weaker (*p* > 0.08, all tests). In late life, there was a trend that higher BMI (ages 71 years: OR 0.95, *p* = 0.08, model 3), was associated with a decreased likelihood of being β-amyloid positive (Table 2). Consistent with this, greater increases in BMI between the home visit at age 69 years and Insight 46 assessment at age 71 years were associated with a significantly decreased likelihood of being β-amyloid positive (Table 4). Alternatively, a 1 SD decrease in BMI was associated with an increased likelihood of being β-amyloid positive (OR 1.45, 95% CI 1.09, 1.92). Further adjustment for time between assessments (mean 1.2; SD 0.6 years), presence of significant affective symptoms and exclusion of individuals with MCI did not alter the association (data not shown). There was no evidence of an interaction with *APOE*-ε4 carrier status in any analyses (all interaction *p* values > 0.23).

Being obese at age 36 years was associated with 0.167 ml larger mean HV at age 71 years, although it should be noted that this was a small group (*n* = 13) and confidence intervals were wide. Subsequently, from age 60–64 years onwards, being overweight (*p* < 0.026, all tests, model 3), and to a lesser extent, obese (*p* < 0.28, all tests, model 3), was associated with larger HV compared with being normal weight (Table 3). The association at age 36 years was attenuated when adjusting for WBV (*p* = 0.12), but all other associations remained largely unchanged. Exclusion of individuals with MCI did not substantially alter associations observed from age 60–64 years onwards (data not shown). BMI changes were not associated with mean HV (Table 4).

In single time-point (continuous and categorical) and change analyses, there was no clear evidence of sex interactions for WMHV, WBV or amyloid analyses (all sex interactions *p* > 0.05). In hippocampal analyses, there was a suggestion of a sex interaction in continuous BMI analyses at age 69 (*p* = 0.038) with a similar, albeit borderline, interaction at age 71 years (*p* = 0.055), suggesting that higher BMI was associated with larger HV in women, but not men (age 69: women: coefficient 0.009 ml per kg/m^2^, 95% CI 0.001, 0.016, *p* = 0.027; men: − 0.005 ml per kg/m^2^, 95% CI -0.015, 0.005, *p* = 0.36; age 71: women: 0.009 ml per kg/m^2^, 95% CI 0.001, 0.017, *p* = 0.028; men: − 0.003 ml per kg/m^2^, 95% CI -0.013, 0.006, *p* = 0.50); there was however no evidence of a similar interaction in the categorical analyses (all interaction *p* values > 0.09).

Removing underweight individuals did not alter categorical weight analysis results.

There were no associations between AC and AC change at any age and WMHV, amyloid status or mean HV. Coefficients did not substantially alter between models and therefore results for model 3 only are presented (Tables 5 and 6). There was no association between AC at any time point and WBV, but increasing AC between 60–64 and 69 years was associated with smaller WBV (model 3: *p* = 0.010, Table 6). In the amyloid analyses, there was no evidence of an *APOE*-ε4 interaction (all interaction *p* values > 0.20). There was no evidence of sex interactions in AC analyses (all interaction *p* values > 0.08).

**Table 5:** Associations between abdominal circumference across adulthood, and global WMHV, β-amyloid status and brain volumes at age 71

	Relative change in WMHV (95% CI)	β-amyloid adjusted OR (95% CI)	WBV ***β*** coefficient (95% CI)	Mean HV ***β*** coefficient (95% CI)
	Model 3	Model 3	Model 3	Model 3
**36 years** WMHV: Model 3: *n* = 409 Amyloid: Model 3: *n* = 412 WBV/HV: Model 3: *n* = 401	0.91 (0.81, 1.02)	1.08 (0.80, 1.47)	4.0 (−1.0, 9.1)	0.018 (− 0.014, 0.051)
**43 years** WMHV: Model 3: *n* = 425 Amyloid: Model 3: *n* = 431 WBV/HV: Model 3: *n* = 417	0.95 (0.84, 1.06)	0.91 (0.66, 1.25)	2.5 (− 2.7, 7.7)	0.011 (− 0.022, 0.044)
**53 years** WMHV: Model 3: *n* = 437 Amyloid: Model 3: *n* = 442 WBV/HV: Model 3: *n* = 429	0.93 (0.84, 1.03)	0.88 (0.67, 1.15)	1.5 (− 2.8, 5.9)	0.003 (− 0.026, 0.032)
**60/64 years** WMHV: Model 3: *n* = 448 Amyloid: Model 3: *n* = 454 WBV/HV: Model 3: *n* = 440	0.95 (0.87, 1.05)	0.80 (0.62, 1.03)	0.0 (− 4.0, 4.0)	0.007 (− 0.019, 0.033)
**69 years** WMHV: Model 3: *n* = 447 Amyloid: Model 3: *n* = 449 WBV/HV: Model 3: *n* = 439	0.97 (0.88, 1.06)	0.83 (0.65, 1.05)	− 1.8 (− 5.5, 2.0)	0.004 (− 0.020, 0.029)

All coefficients or ORs quoted are per 10 cm increase in AC. Associations with WMHV were investigated using GLM, amyloid status using logistic regression and brain volumes using linear regression models. 95% confidence intervals are also shown. Associations significant at *p* < 0.05 are highlighted in bold. Model numbers are stated in the left-hand column. Results are similar across models and therefore only results for fully-adjusted model 3 are presented. Model 3 analyses adjust for sex, TIV, scanning age, contemporaneous SBP, DM, hypercholesterolaemia, adult SEP, smoking (WMHV models) and also β-amyloid status and global WMHV (brain volume analyses). β-amyloid model 3 analyses adjust for sex, contemporaneous SBP and APOE-ε4 status. *AC* abdominal circumference, *CI* confidence interval, *DM* diabetes mellitus, *GLM* generalised linear model, *SBP* systolic blood pressure, *SEP* socioeconomic position, *TIV* total intracranial volume, *WMHV* white matter hyperintensity volume

**Table 6:** Associations between abdominal circumference change and global WMHV, β-amyloid status and brain volumes at age 71

	Relative change in WMHV (95% CI)	β-amyloid adjusted OR (95% CI)	WBV ***β*** coefficient (95% CI)	Mean HV ***β*** coefficient (95% CI)
	Model 2(c)	Model 2(c)	Model 2(c)	Model 2(c)
**36–43 years** WMHV: Model 2: n = 390 Amyloid: Model 2: *n* = 391 WBV/HV: Model 2: *n* = 382	0.98 (0.89, 1.09)	0.95 (0.72, 1.26)	1.4 (− 3.2, 5.9)	0.006 (− 0.023, 0.035)
**43–53 years** WMHV: Model 2: *n* = 391 Amyloid: Model 2: *n* = 391 WBV/HV: Model 2: *n* = 383	0.96 (0.86, 1.08)	0.89 (0.67, 1.17)	−1.4 (− 6.0, 3.3)	− 0.013 (− 0.042, 0.017)
**53–60/64 years** WMHV: Model 2: *n* = 394 Amyloid: Model 2: *n* = 395 WBV/HV: Model 2: *n* = 386	1.01 (0.90, 1.13)	0.84 (0.63, 1.10)	− 1.7 (− 6.3, 2.9)	0.007 (− 0.022, 0.036)
**60/64–69 years** WMHV: Model 2: *n* = 395 Amyloid: Model 2: *n* = 396 WBV/HV: Model 2: *n* = 387	1.00 (0.89, 1.12)	0.95 (0.73, 1.25)	**− 6.0 (− 10.5, − 1.5)**	− 0.013 (− 0.042, 0.016)

WMHV coefficients represent the relative change in mean WMHV per 1 SD increase in the expected AC change across each time-interval using GLM. Adjusted amyloid ORs are quoted per 1 SD increase in the expected BMI change from logistic regression models. Brain volume β coefficients represent the change in brain volume (ml) per 1 SD increase in the expected AC change from linear regression models. Associations significant at *p* < 0.05 are highlighted in bold. Results are similar across models and therefore only results for fully-adjusted model 2(c) are presented. Model 2(c) analyses examine each change variable separately and adjust for sex, TIV, scanning age, contemporaneous SBP, DM, hypercholesterolaemia, adult SEP, smoking (WMHV models) and β-amyloid status and global WMHV (brain volume analyses). β-amyloid model 2(c) analyses examine each change variable separately and adjust for sex, contemporaneous SBP and APOE-ε4 status. *AC* abdominal circumference, *CI* confidence interval, *DM* diabetes mellitus, *GLM* generalised linear model, *HV* hippocampal volume, *OR* odds ratio, *SBP* systolic blood pressure, *SD* standard deviation, *SEP* socioeconomic position, *TIV* total intracranial volume, *WBV* whole brain volume, *WMHV* white matter hyperintensity volume

There was no evidence that duration of obesity was associated with imaging outcome measures (WMHV exponentiated coefficient 0.98, 95% CI 0.92, 1.05; adjusted β-amyloid odds ratio 0.96, 95% CI 0.81, 1.13; WBV *β* coefficient 0.3, 95% CI − 2.5, 3.0; mean HV *β* coefficient 0.008, 95% CI − 0.009, 0.026).

With respect to included vascular covariates in fully-adjusted models, using the continuous BMI analysis at age 71 years as a representative model: contemporaneous SBP was associated with larger WMHV (exponentiated coefficient 1.01, 95% CI 1.002, 1.01, *p* = 0.007), being diabetic was associated with smaller WBV (*β* coefficient − 18.8, 95% CI − 32.2, − 5.3, *p* = 0.006) and ex-smokers had significantly smaller HV compared with non-smokers (*β* coefficient − 0.07, 95% CI − 0.12, − 0.009, *p* = 0.024). Cholesterol status was not associated with any imaging measure (*p* > 0.61, all models).

## Discussion

In this population-based cohort, all of very similar age at the time of assessments, we did not observe an association between higher or increasing adiposity, as measured using BMI, or overweight/obese status, during early adulthood, midlife and early late life, and WMHV (a marker of cerebral SVD) or whole brain volume at age 71 years. Conversely, higher BMI from midlife was associated with decreased likelihood of being β-amyloid positive, and having larger mean hippocampal volume. Declining BMI in the year prior to scanning was particularly associated with increased likelihood of β-amyloid positivity. There was no evidence that cumulative exposure to obesity was associated with brain structure and pathology in early late life. Additional post hoc analysis identified no significant association between cognitive function (PACC) at age 69 and BMI at ages 60, 69 or 71.

Possible mechanisms by which obesity has been suggested to influence cerebrovascular disease include via its relationship with other vascular risk factors such as hypertension, insulin resistance and hyperlipidaemia [11]. However, adjustment for vascular risk factors cannot explain the lack of association in our analysis because we did not see an association between increased adiposity (using both BMI and also AC: a measure of central adiposity, thought to be a better marker of more metabolically active visceral fat) and higher burdens of WMH in either adjusted or unadjusted analyses. Previous population-based studies investigating the relationship between elevated adiposity and cerebral SVD have not, in general, reported significant associations. Neither the AGES-Reykjavic study [27], Framingham cohort [28] nor the ARIC study [29] found an association between adiposity in midlife and subsequent WMHV. A further cross-sectional study in the Framingham cohort failed to find an association between WMH and obesity using visceral and subcutaneous fat measured by CT [30]. In contrast, the CAIDE study reported an association between both midlife and late-life obesity and late-life WMH, even accounting for other vascular risk factors. The risk however was mitigated in those who lost weight by late life [31], which may suggest the midlife association was driven by the tendency for BMI to track over time, and risk accumulation may be critical. We however did not find evidence to support this theory.

Overweight, but not obese, individuals, at age 53 were less likely to be β-amyloid positive at age 71 years, but there was no relationship between midlife continuous measures of adiposity (BMI/AC) and subsequent β-amyloid status, and therefore this finding should be treated with caution. Being obese in midlife was not adversely associated with β-amyloid status at age 71 years, which is in contrast to findings reported by the ARIC study [32]. This might be a consequence of the older population investigated in ARIC, and a higher prevalence of obesity in midlife (25.8%), compared with 17.8% in the 1946 cohort at age 53 years. The Mayo Clinic Study of Aging however failed to observe an association between midlife obesity and later-life β-amyloid status, despite having a higher proportion of midlife obesity (33%) [33].

In early late life (ages 69 and 71 years), there was a trend towards lower BMI being associated with a greater likelihood of being β-amyloid positive; in keeping with this, those individuals who had a decelerating/declining BMI trajectory in the 1–2 years prior to imaging were more likely to be β-amyloid positive. It is well reported that BMI declines in the years prior to clinically manifest dementia, both all-cause, and clinically diagnosed AD dementia [34, 35], the so-called obesity paradox, thought to reflect reverse causality [8–10]. In preclinical AD, an inverse relationship between BMI and β-amyloid burden has been reported in ADNI subjects, although they did not observe a change in BMI longitudinally between β-amyloid positive and negative individuals [36]. Our approach, which assesses how an individual’s trajectory has changed compared with what would be expected based on the previous trajectory, is likely to be more sensitive to early changes in slope rather than assessing absolute value change. The Harvard Aging Brain study also reported an inverse relationship between BMI and β-amyloid burden, but, unlike in our study, only in *APOE*-ε4 allele carriers [37].

Possible mechanisms linking changes in body composition with AD pathology include β-amyloid adversely influencing hypothalamic satiety mechanisms. Corticolimbic structures are involved in modulating hypothalamic control of food intake, including the orbitofrontal cortex and the cingulate cortex [38], both areas affected by early β-amyloid deposition [39]. β-amyloid pathology within the hypothalamus may also disrupt leptin (an appetite-regulating adipokine)-mediated metabolic control [40]. Neuropsychiatric changes such as depression may precede cognitive symptoms in the preclinical phase [41] influencing dietary behaviour, although including a measure of affective symptoms in the analysis did not influence the relationship, arguing against this possibility. Alternatively, physical frailty, a phenomenon in older age which includes reduced gait speed and reduction in BMI, and β-amyloid accumulation, may share a common underlying pathophysiological mechanism, such as inflammation [42].

We found no associations between BMI, BMI change or overweight/obese status across adulthood and later-life whole brain volume. However, increasing central adiposity, from age 60/64 to 69 years was associated with smaller WBV, although this did not reach significance in the single time-point analyses. The lack of a similar finding in BMI work may be because central adiposity is a better marker of visceral adiposity, which is more metabolically active than subcutaneous fat. Obesity is associated with increased production of pro-inflammatory cytokines, such as IL-6 and TNFα, which are associated with cognitive decline [43]. However, a similar association was not observed when examining relationships with mean hippocampal volume meaning this finding should be treated with caution.

However, from late midlife, being overweight, and latterly, obese, was positively associated with mean hippocampal volume at age 71 years, with a similar trend in the continuous BMI analyses, even accounting for head size. These associations were independent of β-amyloid pathology and WMHV, and a sensitivity analysis which excluded individuals with MCI did not reduce this association. Furthermore, reverse causality is unlikely to entirely account for the association since it extended back ~ 8 years prior to scanning in a dementia-free cohort. Interestingly, this is consistent with a previous finding from the NSHD that individuals with weight gain at age 53 years had better memory function at that age [44]. It is possible that the association, to a degree, represents a *selective* protective effect of increased adiposity on hippocampal volume, which would explain the discrepancy with WBV findings. Leptin, produced by adipose tissue, has been shown to have an acute neurotrophic and neuroprotective effect on the hippocampus [45]. However, although circulating leptin levels are higher in obesity, CSF levels have been shown to be reduced, suggestive of a central resistance [46], and would argue against this hypothesis, and a similar relationship was not observed in the central adiposity analyses. This observation requires further investigation in longitudinal imaging work.

Several large population-based studies have investigated the relationship between midlife adiposity and brain volume with inconsistent findings. The Framingham Offspring Cohort found an inverse cross-sectional relationship between measures of adiposity, particularly visceral fat, and total brain volume in midlife, and larger temporal horn volume (THV) (a proxy marker of hippocampal volume) with greater waist:hip ratio (WHR), accounting for vascular risk factors. Longitudinally they found an inverse relationship between midlife WHR, but not BMI, and longitudinal global brain loss but no relationship with THV [28]. More recently, the AGES-Reykjavik study found no association between midlife higher adiposity and late-life total brain volume [27]. Cross-sectional studies in late life have also reported a negative relationship between higher BMI and global brain volume [47]. None to our knowledge has reported positive associations between BMI and brain volumes. Our findings might reflect a survival bias in this study, whereby overweight/obese individuals who were susceptible to the negative impact of obesity on neuronal health have been lost to follow up.

A relationship between obesity, particularly in midlife and late-life dementia risk, has been reported across several population studies, although this is not consistently the case [8]. A large population-based study in the UK reported an inverse association between midlife obesity and late-life dementia risk, and a corresponding positive association with midlife underweight status [48], which is more consistent with our findings. There is limited literature on adiposity trajectories and their association with dementia risk. The Honolulu-Asia Aging study (HAAS) failed to find a relationship between BMI trajectories in men and late-life all-cause dementia, but greater BMI increases were seen in individuals who developed clinically-diagnosed vascular dementia, even accounting for other vascular risk factors [49]. In contrast, a study in Swedish women found slower BMI increases from 38 to 70 years in those who went on to develop dementia [50]. Discrepancies between studies may arise due to population differences (including sex, age at baseline and study duration) and methodological differences (including approaches to measuring adiposity and number/timing of measurements collected).

This study has several strengths, including the multiple time-points at which adiposity metrics have been measured, the very similar age of participants, who are broadly representative of people born in mainland Britain in 1946, and use of a single scanner.

### Limitations

Limitations include the possibility of survival bias, and loss of individuals with pre-existent significant cognitive symptoms, which may mask possible associations between obesity and cerebral pathology. There are limitations inherent to any birth cohort. Whilst participants are broadly representative of the population born in mainland Britain in 1946, Insight 46 is a cohort consisting of exclusively white British participants, which might reduce generalisability to non-white populations. Moreover, having all been born in the same week, participants went through childhood, adolescence and midlife at the same time and are likely to have been exposed to the similar environmental and societal factors, and prior to current guidance and advice regarding weight, diet, and exercise. These factors are likely to differ from those of individuals born at other times. Individuals in Insight 46 tended to have lower BMI and AC than those in the larger NSHD cohort, although absolute differences were small. We have previously demonstrated that Insight 46 participants are healthier with lower rates of overweight/obesity at age 69 years than in the larger NSHD cohort, and, consistent with these findings, obese individuals were less likely to tolerate scanning [15] reducing the ability to detect true associations. Rates of dementia at this age are very low – 3/471 individuals in this study were diagnosed with dementia and excluded (Figure e**1**), and we do not think these are likely to have affected our results. There are few underweight individuals, limiting the power to detect potential U-shaped relationships between adiposity and late life cerebral pathology. Obesity may have a detrimental impact on other markers, such as lacunes (noting their relatively low prevalence in our cohort ~ 7%), which we did not investigate. Imaging was only available at a single time-point, and therefore it was not possible to determine the influence of adiposity on longitudinal imaging changes: this will be addressed in future work. Furthermore, because this cohort is largely cognitively normal, we cannot directly investigate associations with dementia prevalence at the present time.

In conclusion, we did not find consistent associations to explain the reported relationship between obesity, particularly in midlife, and late-life dementia risk, using WMHV, β-amyloid status and brain volumes as indicators of brain health. Indeed, being overweight or obese in later midlife and early late life was associated with larger hippocampal volumes, and declining BMI in the 1–2 years prior to scanning was associated with increased risk of β-amyloid positivity, which may reflect the influence of neurodegeneration on body composition. Our findings do not support interventions to tackle obesity as an effective approach towards improving later-life cerebral health, although these remain important for improving other health outcomes including cardiovascular and cancer risk. Declining BMI in later life may be a marker of preclinical AD.

## Supplementary Information

**Additional file 1.**

## Data Availability

A data-sharing policy is in place: anonymised data will be shared by request from any qualified investigator (https://skylark.ucl.ac.uk/NSHD/doku.php).
