# Supplementary material for: Investigating the relationship between BMI across adulthood and late life brain pathologies
Source: Alzheimers Res Ther. 2021 Apr 30;13:91. doi: 10.1186/s13195-021-00830-7 (PMC8091727; doi:10.1186/s13195-021-00830-7)
Supplement: Supplementary file 1 — Additional file 1. [file 13195_2021_830_MOESM1_ESM.pdf]

Investigating the relationship between BMI across the life course and late life brain pathologies – Supplementary material

eMethods

Figure e-1 Flowchart providing an overview of Insight 46 recruitment from the MRC NSHD (modified with permission from James *et al*[1] under the terms of the Creative Commons Attribution 4.0 International License (http://creativecommons.org/licenses/by/4.0/)) and summary of imaging data available. BaMoS, Bayesian Model Selection; MRI, magnetic resonance imaging; MCI, mild cognitive impairment; NSHD, National Survey of Health and Development; PET, positron emission tomography; QC, quality control; WMHV, white matter hyperintensity volume.

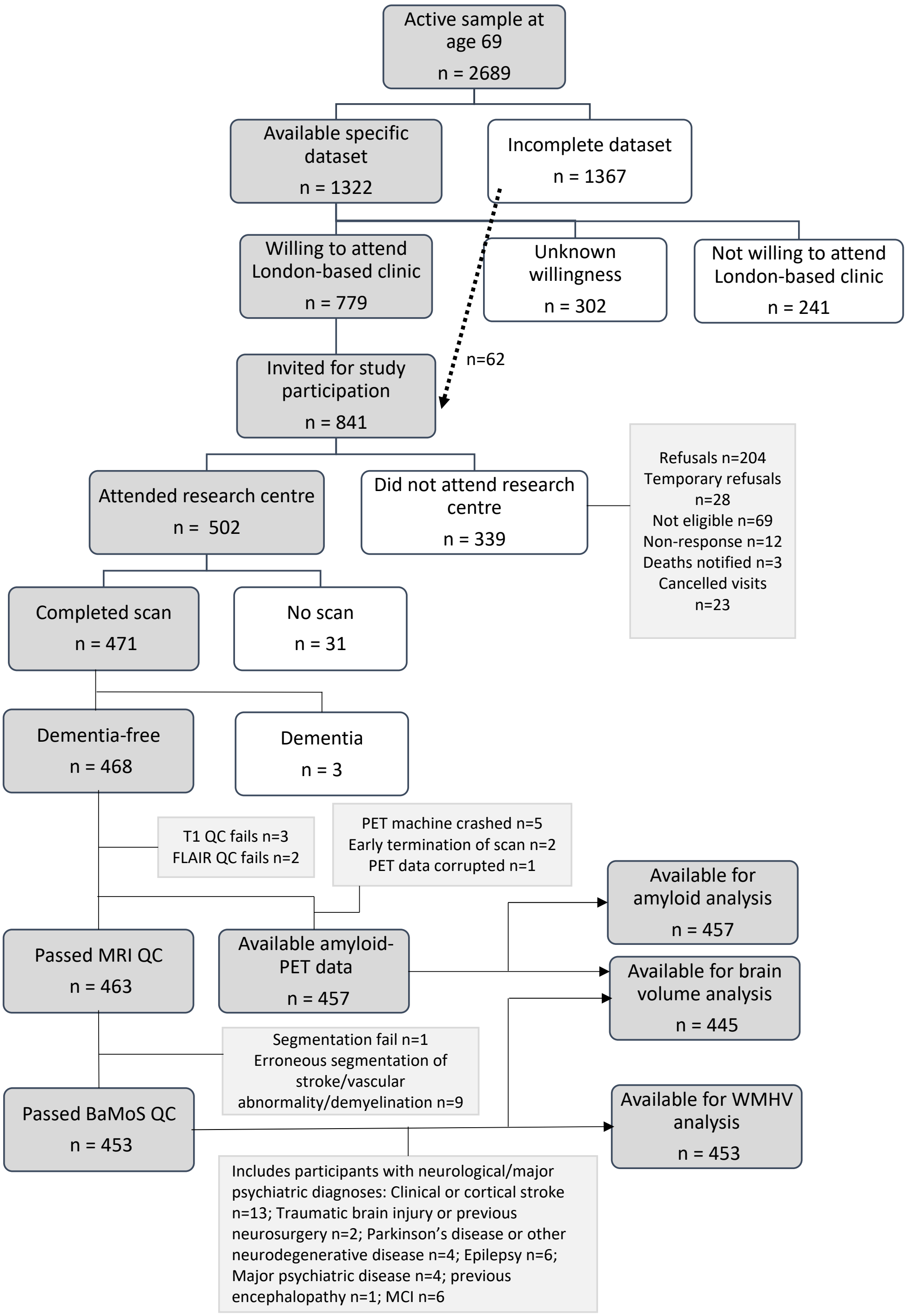

eResults

Table e-1 Comparison of clinical characteristics between individuals with missing imaging and BMI data and those who completed scanning and had complete BMI data. By design, no individuals were missing BMI information at all time-points. 54 individuals were missing a single measurement, 9 individuals were missing a BMI measurement at two time points and 1 individual was missing a measurement at three time points. 2 individuals were missing *APOE*-ε4 data. Specific *n* are provided for a given variable. BMI, body mass index; BP, blood pressure; IQR, interquartile range; MMSE, mini-mental state examination; n, number; NA, not applicable; SBP, systolic blood pressure; SD, standard deviation

Variable		Complete BMI information and completed scanning (n=404)	Missing BMI at any age but completed scanning (n=64)	Did not complete scanning protocol (n=31)
**Male:Female**		208:196	32:32	15:16
**Age at Insight 46 assessment, mean (SD)**		70.7 (0.7)	70.8 (0.7)	70.8 (0.6)
**Amyloid positive, n (%)**		71 (18.0) n=394	12 (19.1) n=63	NA
**Whole brain volume in ml, mean (SD)**		1101.3 (97.9) n=383	1091.8 (101.7) n=62	NA
**Mean hippocampal volume in ml, mean (SD)**		3.1 (0.3) n=383	3.1 (0.4) n=62	NA
**White matter hyperintensity volume in ml, median (IQR)**		3.3 (1.7, 6.9) n=391	2.3 (1.0, 5.2) n=62	NA
**Total intracranial volume in ml, mean (SD)**		1434.9 (132.2) n=391	1428.2 (133.6) n=62	NA
**MMSE /30, mean (SD)**		29.3 (0.9)	29.2 (0.9)	29.4 (1.0)
**BMI at 36 in kg/m^2, mean (SD)**		23.7 (3.1)	23.5 (2.9) n=25	24.8 (3.6) n=30
**BMI at 43 in kg/m^2, mean (SD)**		24.9 (3.2)	24.8 (3.2) n=44	26.5 (4.0)
**BMI at 53 in kg/m^2, mean (SD)**		27.0 (4.1)	26.4 (3.8) n=55	28.1 (4.8) n=30
**BMI at 60-64 in kg/m^2, mean (SD)**		27.6 (4.2)	27.4 (3.9)	29.6 (5.6)
**BMI at 69 in kg/m^2, mean (SD)**		27.6 (4.4)	27.4 (4.2) n=57	30.7 (5.5) n=30
**BMI at 71 in kg/m^2, mean (SD)**		27.5 (4.4)	27.7 (4.8)	30.4 (5.7)
**Smoking Status at age 68, n (%)**	**Current smoker**	14 (3.5)	2 (3.1)	2 (6.5)
	**Ex-smoker**	245 (60.6)	44 (68.8)	22 (71.0)
	**Never smoker**	145 (35.9)	18 (28.1)	7 (22.6)
**Hypercholesterolaemia at age 71, n (%)**		319 (79.0)	52 (81.3)	27 (87.1)
**Diabetes mellitus at age 71, n (%)**		42 (10.4)	8 (13.3) n=60	5 (16.7) n=30
**SBP at age 71 in mmHg, mean (SD)**		137.0 (16.5) n=403	136.9 (19.0)	138.6 (18.3)
**Adult socioeconomic position, n (%)**	**Non-manual (Class I-IIIN)**	354 (87.6)	44 (68.8)	26 (83.9)
	**Manual (Class IIIM-V)**	50 (12.4)	20 (31.3)	5 (16.1)
***APOE*-ε4 carrier (1 or 2 alleles), n (%)**		120 (29.9) n=402	19 (29.7)	7 (22.6)

References

1. James SN, Lane CA, Parker TD, et al. Using a birth cohort to study brain health and preclinical dementia: Recruitment and participation rates in Insight 46. *BMC Res Notes*. 2018; 11(1):885
